# Outer‐sphere residues influence the catalytic activity of a chalcone synthase from *Polygonum cuspidatum*

**DOI:** 10.1002/2211-5463.12072

**Published:** 2016-05-16

**Authors:** Yalin Shen, Xing Li, Tuanyao Chai, Hong Wang

**Affiliations:** ^1^ University of Chinese Academy of SciencesBeijingChina; ^2^ State Key Laboratory of Molecular Developmental BiologyInstitute of Genetics and Developmental BiologyChinese Academy of SciencesBeijingChina

**Keywords:** chalcone synthase, *Polygonum cuspidatum*, site‐directed mutagenesis

## Abstract

We have previously cloned a chalcone synthase (PcCHS1) from *Polygonum cuspidatum* and biochemically identified its enzymatic dynamic properties. Here, we found that the outer sphere residues, Q82 and R105, could affect the catalytic activity and product profile of PcCHS1. Both Q82P and R105Q mutations of PcCHS1 could also change the pH dependence activity as well as the product profile of PcCHS1. Moreover, the Q82P/C198F double mutant could rescue the complete loss of enzyme activity caused by the C198F single mutation. Our study demonstrated that these outer‐sphere residues of PcCHS1 play important roles both in structural maintenance and enzyme activity.

**Abbreviations**
2‐PS: 2‐pyrone synthase
ACS: acridone synthase
BAS: benzalacetone synthase
BNY: bis‐noryangonin
BPS: benzophenone synthase
CTAL: *p*‐coumaroyltriacetic acid lactone
HPLC: high performance liquid chromatography
LC–MS: liquid chromatography–mass spectrometry
OKS: octaketide synthase
OLS: olivetol synthase
PBS: phosphate buffer saline
PcCHS1: *Polygonum cuspidatum* chalcone synthase 1
PCS: pentaketide chromone synthase
PKS: polyketide synthases
SDS/PAGE: sodium dodecyl sulfate polyacrylamide electrophoresis
STS: stilbene synthase

The chalcone synthase (CHS) superfamily of type III polyketide synthases (PKSs) are crucial enzymes responsible for the formation of numerous structurally diverse and biologically significant secondary metabolites, and some of the metabolites can serve as antibiotics, anticancer drugs, and immunosuppressants 1, 2, 3, 4. CHS and homologous members of type III PKSs are usually homodimers of 40–45 kDa subunits, and they perform a complete series of decarboxylation, condensation, cyclization, and aromatization reactions in a single active site 5. The differences in reactions catalyzed by various type III PKSs mainly include the selection of the starter substrates, the number of condensation reactions, and the type of ring closure of the products 6, 7. CHS (EC 2.3.1.74) was the first discovered type III PKS due to its important role in the first committed step in flavonoid biosynthesis by catalyzing consecutive decarboxylative condensations of *p*‐coumaroyl‐CoA or other analogs with three C2 units from malonyl‐CoA and subsequent cyclization of the linear tetraketide intermediate to form chalcone, the precursor of diverse flavonoids 2 (Fig. 1).

**Figure 1:**
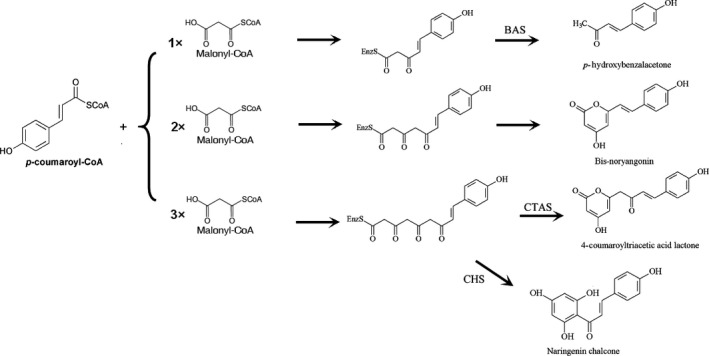
Reactions and products catalyzed by CHS, BAS, and *p*‐coumaroyl triacetic acid synthase (CTAS).

A growing number of type III *PKSs* genes have been cloned and identified from various plants, including *ACS* 8, *STS* 9, *2‐PS* 10, *BAS* 11, *BPS* 12, *OLS* 13, *QNS* 14 and so on, and it is interesting that they share a similar three‐dimensional overall structure and catalytic machinery, and contain a conserved Cys164‐His303‐Asn336 catalytic triad [numbering in *Medicago sativa* CHS (MsCHS)] in an internal active‐site 7. It has been shown that merely a subtle modification of the volume and shape of the active‐site cavity could result in a remarkable change in enzyme function. A few studies referring to CHS and other enzymes have also indicated that mutations on nonactive‐site residues sometimes could affect enzyme activity in a direct way 15, 16, 17.

*Polygonum cuspidatum* Sieb. et Zucc. (Polygonaceae) is widely used in China and Japan for the treatment of atherosclerosis, hypertension, cough, suppurative dermatitis, and gonorrhea, and it is also rich in aromatic polyketides such as anthraquinones and stilbenes 18. Some *PKSs* genes, including *PcPKS1* 19, *PcPKS2* 20, *PcCHS1* 21, *PcPKS3,* and *PcPKS5* (*PcSTS*) 22, have been cloned recently in our lab to investigate their possible roles in the biosynthesis of those aromatic polyketides, among these genes, *PcCHS1* was phylogenetically clustered in CHS group. The catalytic triad of Cys164, His303, and Asn336 (numbering in MsCHS), the CHS ‘gatekeeper’ Phe215 and Phe265 2, and CoA‐binding residues, were all conserved in PcCHS1 (Fig. 2). However, Thr197 which plays a pivotal role for the chalcone‐forming activity in *M. sativa* CHS 23, 24, 25 was replaced with Cys198 in PcCHS1. Additionally, P82 and K105 conserved in plant PKSs were substituted by Gln and Arg in PcCHS1, respectively (Fig. 2). Thus, in this study, we report the influence of two outer‐sphere‐substituted residues, Q82 and R105, and one active‐site‐substituted residue, C198, on the biochemical characteristics of a PcCHS1 from *P. cuspidatum* by site‐directed mutagenesis, and our results suggested that some residues far away from the active‐site of type III PKSs also played important roles in directing enzyme activity and product profiles.

**Figure 2:**
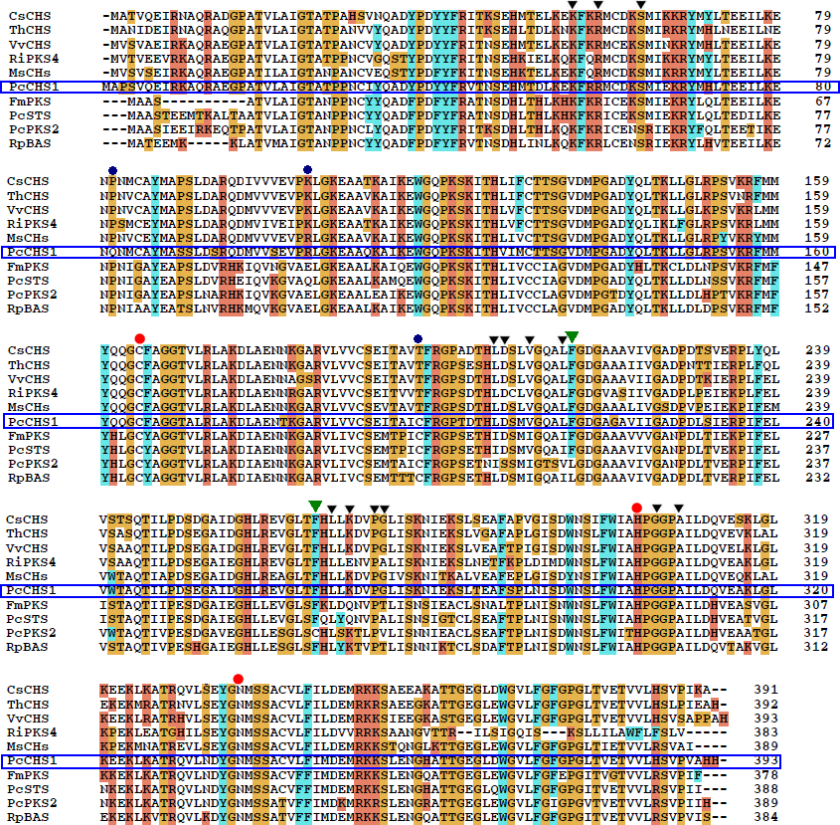
Comparison of primary sequences of *Polygonum cuspidatum* PcCHS1 and other CHS superfamily enzymes. The catalytic residues, Cys164, His303, and Asn336, conserved in the CHS superfamily enzymes are marked with 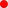, and residues for CoA binding with ▼. CHS ‘gatekeepers’, Phe215 and Phe265 are marked with 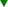. Residues which performed site‐directed mutagenesis in PcCHS1 are marked with 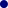. The abbreviations for species are: PcPKS2 (*P. cuspidatum* PKS2), MsCHS (*Medicago sativa* CHS), RiPKS4 (*Rubus idaeus* PKS4), PcSTS (*P. cuspidatum* STS), VvCHS (*Vitis vinifera* CHS), CsCHS (*Camellia sinensis* CHS), ThCHS (*Tricyrtis hirta* CHS), FmCHS (*Fallopia multiflora* CHS), and RpBAS (*Rheum palmatum* BAS).

## Materials and methods

### Plant material and chemicals

*Polygonum cuspidatum* Sieb. et Zucc. plants were maintained in a greenhouse at the Institute of Botany, the Chinese Academy of Sciences, Beijing, China. The plants' leaves were frozen in liquid nitrogen and stored at −80 °C immediately after harvesting.

Malonyl‐CoA, benzoyl‐CoA, acetyl‐CoA, and naringenin were purchased from Sigma (St. Louis, MO, USA). *p*‐hydroxybenzalacetone (*p*‐hydroxyphenylbut‐3‐ene‐2‐one) was purchased from Alfa Aesar GmbH & CoKG (Karlsruhe, Germany). *p*‐coumaroyl‐CoA were synthesized as described in 26.

### Sequence analysis and homology modeling

Multiple alignments of amino acid sequences were performed using Clustal W 27. Homology modeling was performed using swiss‐pdb viewer 28. The corresponding Ramachandran plot was also created with swiss pdb viewer software that the majority of residues were confirmed grouped in the energetically allowed regions. The cavity volumes were calculated by the program castp (http://cast.engr.uic.edu/cast/). All protein structure figures were prepared with pymol (DeLano Scientific, http://www.pymol.org).

### Heterologous expression and purification of recombinant PcCHS1 and its mutants

The bacterial expression vector pET‐30a (+) containing the ORF of wild‐type *PcCHS1*, as well as its mutants, was transformed into *Escherichia coli* BL21‐Rosetta (DE3) (TransGen, Beijing, China), then grown at 220 r.p.m. and 37 °C in 200 mL of Luria–Bertani (LB) medium containing kanamycin (50 μg·mL^−1^). When the absorbance at 600 nm reached 0.6–0.8, 1 mm IPTG was added and then incubated at 28 °C for 8 h. After being harvested by centrifugation, the *E. coli* cells were resuspended in 3 mL of 0.1 m PBS buffer (pH 7.5), and then sonicated on ice for 10 min. The homogenate was centrifuged at 12 000 ***g*** for 15 min at 4 °C. The supernatant was passed through a column of Ni‐nitrilotriacetic acid His‐Bind^™^ Resin (Novagen, Shanghai, China) containing Ni^2+^ as an affinity ligand since the recombinant enzyme contained a hexahistidine tag at the C terminus. After washing with 0.1 m PBS buffer (pH 7.5) containing 0.5 m NaCl and 40 mm imidazole, the recombinant PcCHS1 and its mutants were eluted with 0.1 m PBS buffer (pH 7.5) containing 400 mm imidazole. For long‐term storage, the buffer was changed to 0.1 m PBS buffer (pH 7.5) containing 10% (v/v) glycerol using PD‐10 columns (Amersham Pharmacia Biotech, Uppsala, Sweden), and the sample was stored at −80 °C. The efficiency of purification was detected by SDS/PAGE. Protein concentration was determined by the Bradford method with bovine serum albumin as the standard.

### Enzyme reaction and product analysis

The standard reaction system and product analysis were similar to those by Ma *et al*. 20. Quantitative determinations were carried out using Agilent 1100 HPLC system on an Agilent TC‐C18 reverse phase column (5 μm, 250 mm × 4.6 mm; Macherey Nagel, Düren, Germany). For the standard assay, gradient elution was performed with H_2_O and MeOH at a flow rate of 1 mL·min^−1^, with 20% MeOH for 0–2 min; 20–70% MeOH for 2–12 min; 70–80% MeOH for 12–14 min; 80–95% MeOH for 14–16 min; 95% MeOH for 16–20 min; and 95–20% MeOH for 20–23 min. Retention time was: *p*‐hydroxybenzalacetone for 10.07 min, *p*‐coumaroyltriacetic acid lactone (CTAL) for 11.3 min, bis‐noryangonin (BNY) for 11.6 min, and naringenin for 12.6 min. The detection wavelengths were 289 nm (naringenin), 323 nm [*p*‐hydroxybenzalacetone (BA); CTAL], and 365 nm (BNY).

### Enzyme kinetics

Steady‐state kinetics parameters were determined using five concentrations covering the *K*_m_ range of 0.5–5.0 of one substrate with excessive amount of the other. The experiments were carried out in triplicate. The kinetic constants were calculated for formation of the major product at the optimum pH. Lineweaver–Burk plots of the data were employed to derive the apparent *K*_m_ and *k*_cat_ values.

### Site‐directed mutagenesis

Mutations (Q82P, C198F, Q82P/C198F, and R105Q) were introduced into *PcCHS1* cloned in pET30a(+) using the QuikChange site‐directed mutagenesis kit (Stratagene, Shanghai, China) with the following primers: Q82P sense: 5′–GGA GAT CCT CAA GGA GAA CCC AAA CAT GTG TG–3′, antisense: 5′–GGA GAT CCT CAA GGA GAA CCC AAA CAT GTG TG–3′; R105Q sense: 5′–GTG GTG AGT GAG GTG CCA CAG CTC GGC AAA–3′, antisense: 5′–TGT GGC ACC TCA CTC ACC ACC ATA TCC TGC–3′; C198F sense: 5′–ATC ACG GCT ATT TCC TTC CGT GGG CCG ACA G–3′, antisense: 5′–TCG GCC CAC GGA AGG AAA TAG CCG TGA TCT C–3′. After confirmation by sequencing, the plasmids that expressed the mutant proteins were transformed into *E. coli* BL21‐Rosetta (DE3) (TransGen), and the respective recombinant enzymes were expressed and purified using the same procedure as that for the wild‐type PcCHS1.

## Results

The cDNA (GenBank accession number: JQ654448) of PcCHS1 was previously amplified in our lab 21. The deduced amino acid sequence of PcCHS1 shared 80–99% identity with those of other type III PKSs of plant origin.

**Figure 3:**
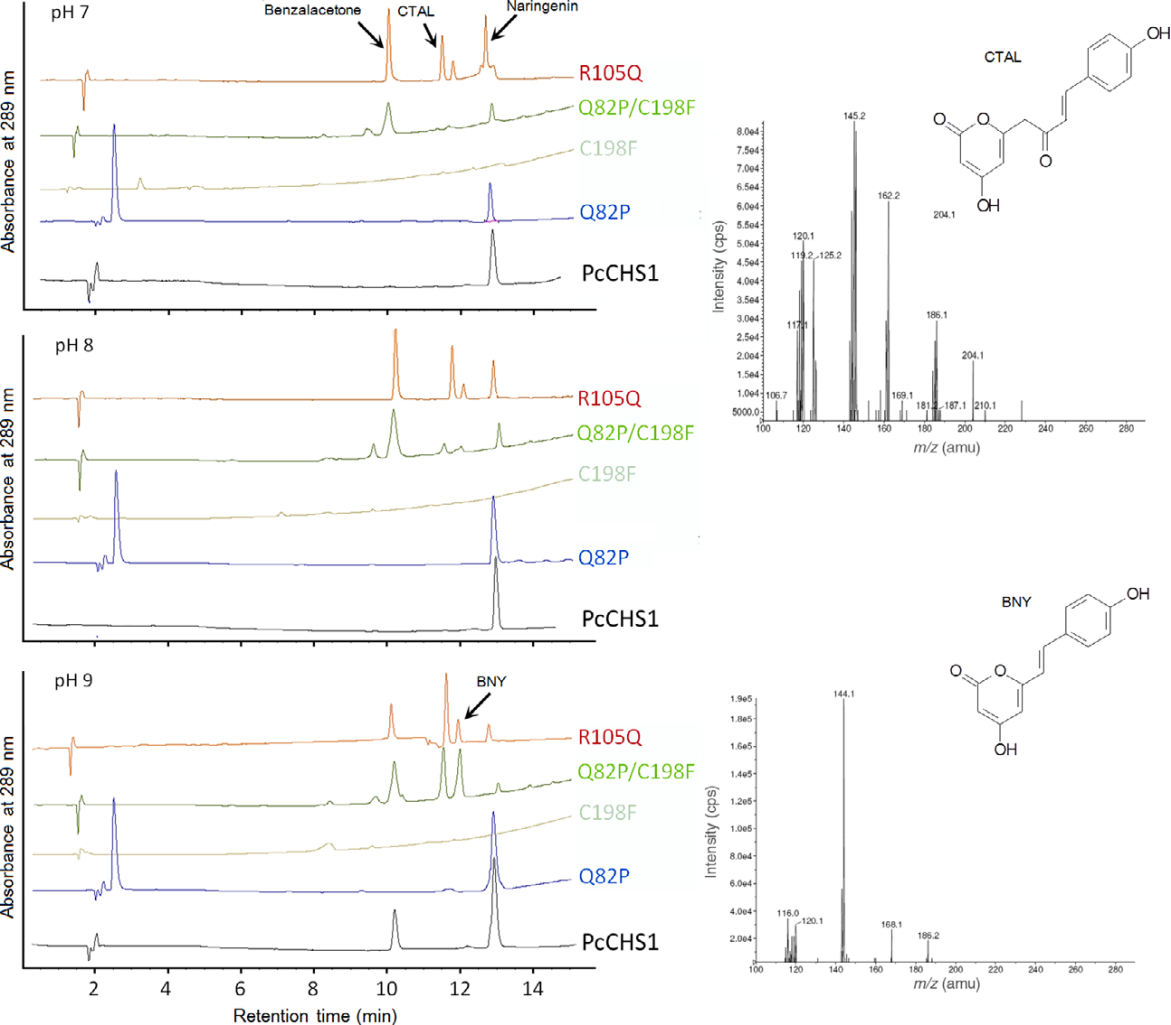
Biochemical characteristics of PcCHS1 and its mutants. HPLC elution profiles of PcCHS1 as well as its mutants (Q82P, R105Q, C198F, and Q82P/C198F) product profiles at pH 7–9 when *p*‐coumaroyl‐CoA was used as a starter substrate. Mass chromatograms of CTAL and BNY were also given. BA and naringenin were identified by comparing the respective retention time with their reference standards.

Identification and quantitation of the enzymatic products by HPLC showed that when incubated with *p*‐coumaroyl‐CoA and malonyl‐CoA at pH 7–8, PcCHS1 catalyzed the formation of naringenin as the major product. When at pH 9, both *p*‐hydroxybenzalacetone and naringenin were detected (Fig. 3). The *K*_m_ and *k*_cat_ values of chalcone‐forming activity, revealed by the steady‐state kinetics analysis at pH 8.0 for *p*‐coumaroyl‐CoA and malonyl‐CoA of PcCHS1, are shown in Table 1.

**Table 1:** Kinetic parameters of the wild‐type and mutants of PcCHS1. Results are means (*n* = 3) with SD < 15%

Enzymes	Products	pH Optimum	*p*‐Coumaroyl‐CoA			Malonyl‐CoA		
			*k*_cat_ (min^−1^)	*K*_m_ (μm)	*k*_cat_/*K*_m_ (m ^−1^·s^−1^)	*k*_cat_ (min^−1^)	*K*_m_ (μm)	*k*_cat_/*K*m (m ^−1^·s^−1^)
PcCHS1	Naringenin	8	6.7	13.6	8210.78	4.9	30.8	2651.52
Q82P	Naringenin	8	3.2	21.3	2503.91	2.3	57.9	662.06
C198F	Almost inactive							
Q82P/C198F	Benzalacetone	8	5.1	19.8	4292.93	3.3	39.7	1385.39
R105Q	Benzalacetone	7	10.7	14.2	12558.69	3.8	37.4	1693.40

### Enzyme activity of PcCHS1 mutants

The mutants of PcCHS1, including single (Q82P, R105Q, C198F) and double (Q82P/C198F) mutants, were successfully constructed according to the protocol of QuikChange site‐directed mutagenesis kit. The mutants of PcCHS1 were heterologously expressed in *E. coli* as the recombinant enzymes with a His tag at each of the C terminuses. Enzyme assays of mutants were carried out similarly as for the wild‐type PcCHS1 with *p*‐coumaroyl‐CoA used as the starter substrate.

The mutant Q82P exhibited reduced chalcone‐forming activity because its *K*_m_ value (Table 1) with *p*‐coumaroyl‐CoA as the starter substrate was about two times higher than that of the wild‐type. Additionally, the Q82P mutant lost *p*‐hydroxybenzalacetone‐forming activity at pH 7–9. The mutant C198F almost completely lost the CHS activity, no product was detected under each pH (Fig. 3). Interestingly, the *p*‐hydroxybenzalacetone and chalcone‐forming activities were restored by the double mutation of Q82P/C198F. The double mutant not only exhibited comparatively high *p*‐hydroxybenzalacetone‐forming activity at pH 8–9 but released also abundant common derailment products, CTAL and BNY, of CHS at pH 9 (Fig. 3). The *K*_m_ value and catalytic efficiency of Q82P/C198F mutant for *p*‐coumaroyl‐CoA are listed in Table 1.

The mutant R105Q changed the pH dependence of *p*‐hydroxybenzalacetone‐forming activity as well as the product profiles at pH 7–9 of wild‐type PcCHS1. At pH 7–8, R105Q mutant catalyzed the formation of four products of common CHS, of which *p*‐hydroxybenzalacetone served as a major product along with CTAL, chalcone, and a little BNY. However, at pH 9, all the products were also produced but in different quantities compared to those produced at pH 7 (Fig. 3). The catalytic efficiency of R105Q mutant for *p*‐coumaroyl‐CoA was higher than that of wild‐type PcCHS1 thanks to its high *k*_cat_ value.

### Homology modeling of PcCHS1

**Figure 4:**
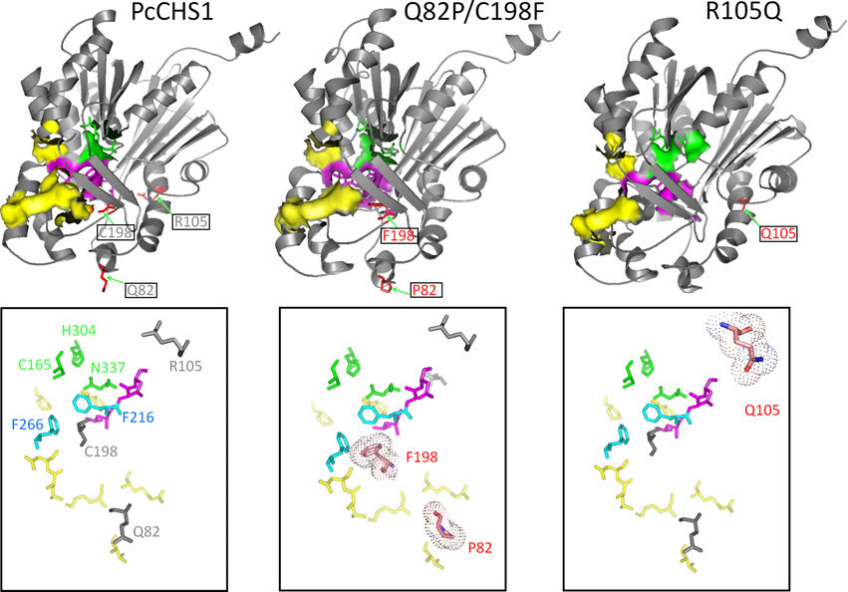
Homology‐modeled overall structure of wild‐type PcCHS1, Q82P/C198F, and R105Q using the original model of MsCHS (PDB code: 1BI5A). The residues mutated in PcCHS1 were shown in sticks with spheres. Residues that constituted the active site, ‘gatekeeper’ phenylalanine, and the substrate‐binding domain were respectively labeled green, blue, and yellow.

Based on the sequence homology of residues 4–390 of PcCHS1 and the X‐ray crystal structures of *M. sativa* CHS (PDB code: 1BI5A), we found they shared 80.9% sequence identity. Then, *M. sativa* CHS was used as a template, and the predicted three‐dimensional structure of PcCHS1 was shown in Fig. 4A by applying a standard homology modeling procedure. The Ramachandran plot of the model showed that 92.8% of the residues were grouped in the most favored regions, 6.3% in the additionally allowed regions, 0.9% in the generally allowed regions, and none in the disallowed regions. The homology model predicted that PcCHS has almost the same overall fold as MsCHS (Fig. 4), and the cavity volume of PcCHS was calculated to be 1076 Å^3^, similar to that (1019 Å^3^) of MsCHS.

## Discussion

So far, five type III PKSs have been isolated and characterized from *P. cuspidatum* in our lab. Functional and enzymatic analyses showed that the recombinant PcPKS1 was a bifunctional enzyme with both CHS and benzalacetone synthase (BAS) activities 19. PcPKS2 was a BAS to produce CTAL, BNY, *p*‐hydroxybenzal‐acetone as major products at pH 7–9 with a trace amount of naringenin 20. PcPKS3 was a CHS to produce naringenin as a major product at pH 7–8 with BNY and CTAL as side products and PcPKS5 was a stilbene synthase (STS) to produce resveratrol as a major product at pH 7 with CTAL and naringenin as side products 22. In many plant‐specific CHS enzyme reactions *in vitro*, BNY and CTAL are usually obtained as early‐release derailment by‐products 29. However, PcCHS1 was unable to yield any obvious side products like CTAL and BNY at pH 6–9 except forming both naringenin and *p*‐hydroxybenzalacetone at pH 9 (Fig. 3). This suggests that PcCHS1 possibly adopts a more ‘stable’ and ‘robust’ structure suitable for its pure chalcone‐forming activity at physiological pH. However, homology modeling did not reveal any special clue about the ‘derailment products‐free’ feature of PcCHS1 (Fig. 4), and sequence analysis also showed that the catalytic triad, CoA‐binding sites, and most active‐site residues, including Thr132, Ser133, Met137, Gly163, Thr194, Val98, Gly211, Phe215, Gly216, Ile254, Phe265, Ser338, and Pro375 (numbering in *M. sativa* CHS), were identical to MsCHS (Fig. 2). Thus, we thought that some other residues surrounding or away from the CoA binding and active‐site cavity might contribute separately or together to the robust chalcone‐forming activity of PcCHS1. In order to verify this supposition, site‐directed mutagenesis experiments were designed. As shown in Fig. 3, when Arg105, a residue on the surface of the protein and far from the CoA binding and active‐site cavity (Fig. 4A), was substituted by Gln, the mutant enzyme largely altered the product profile of the wild‐type PcCHS1 and produced early released derailment side products, BNY and CTAL, at pH 7–9.

In our work, R105 (corresponding to R104 in *M. sativa* CHS) was an outer sphere Arginine in PcCHS1 (Fig. 4A), but not a conserved residue in all CHSs (Fig. 3). In other CHSs, this residue usually appeared as positively charged residues like Arg or Lys; however, in CHS‐like enzymes such as STS and BAS, it acted more like negatively charged residues like Glu or uncharged but polar residues like Gln (Fig. 2). As shown in the results, when R105 was mutated to Gln in PcCHS1, all the side products including *p*‐hydroxybenzalacetone were yielded at pH 7–9 (Fig. 3), and surprisingly, the *p*‐hydroxybenzalacetone‐forming activity of PcCHS1 became stronger than its chalcone‐forming activity. The above results indicated that the ability of PcCHS1 to catalyze cyclization reaction or malonyl‐CoA condensation was somehow damaged in this mutant. Taken together, our results suggested that although remote from the active site, Arg105 played an important role in supporting the structure of PcCHS1 as well as exerting substantial effects on the catalytic activity of the enzyme.

One of the salient characteristic features of PcCHS1 is the replacement of the CHS active‐site residue Thr197 by Cys. Interestingly, the C197 was usually conserved in some CHS‐like PKSs, such as PcSTS and RpBAS, other than in CHSs (Fig. 2). T197 along with other chemically inert residues residing in the active‐site cavity including G256 and V338, are thought to be essential factors for controlling the substrate and product specificity in CHSs. Some mutagenesis works were reported on the role of T197 in enzyme reactions 23, 30, 31. Noel and coworkers found that the *Gerbera hybrida* 2‐pyrone synthase (2‐PS) and MsCHS structures were remarkably similar, except that the active‐site cavity in 2‐PS was smaller due to the increased steric bulk of three active‐site residues, L202, L261, and I343, but the corresponding three counterparts were T197, G256, and S338 in CHS. When they created mutations at these three positions (T197L/G256L/S338I) in CHS, the CHS triple mutant became an enzyme with identical functionality to 2‐PS because it became capable of catalyzing condensation of acetyl‐CoA rather than of bulky *p*‐coumaroyl‐CoA with two molecules of malonyl‐CoA. In addition, the T197L point mutant was shown able to produce a 2‐PS‐like enzyme. This was the first report to show that T197 might directly determine the starter substrate preference by influencing the volume and shape of the active‐site cavity 30. Later, Abe and coworkers performed site‐directed mutagenesis studies on the active‐site residue of M207 in pentaketide chromone synthase (PCS) 31 and of G207 in octaketide synthase (OKS) of *Aloe arborescens* 23 (corresponding to T197 in *M. sativa* CHS), and their results revealed that the 197 residue could also determine the polyketide chain length and the product specificity of the enzyme reaction 7. It is possible that when the mutated 197 residue is not bulky enough to affect the active‐site cavity's accommodating of the preferred starter substrate; it mainly influences the product specificity. Otherwise, it should have changed the starter substrate preference. In our study, when C198 of PcCHS1 was replaced by Phe (the bulk of hydrophobic residues), it failed to show any apparent naringenin and *p*‐hydroxybenzalacetone‐forming activity at pH 8–9. C198F only yielded a trace amount of chalcone and BNY at pH 7 (Fig. 3). The similar reduced activity was observed by G207F mutant in *Aloe arborescens* PCS 23. However, the double mutant, Q82P/C198F, could perform like a common CHS to yield not only chalcone but also its derailment products (Fig. 3). It is more likely the case that by replacing Q82 in C198F mutant of PcCHS1 with the conserved Pro in all the PKSs, the active‐site cavity might be slightly reshaped to restore the p‐hydroxybenzalactone‐ and chalcone‐forming activity. Interestingly since Q82 was on the surface of the enzyme and Q82P produced different product profiles and activities from the wild‐type, the role of Q82 could not be considered simply as maintaining the structural integrity of the enzyme (Fig. 3 and Table 1). In addition, Q82 is too far away from C198 to have any direct interaction with the latter (Fig. 4), which makes the machinery of Q82P to help C198F to restore the CHS activity more complicated to depict.

Taken together, this work reported the biochemical characterization of PcCHS1 from *P. cuspidatum*. We found that the outer‐sphere residues, Q82 and R105, could affect the catalytic activity and product profile of PcCHS1. Both Q82P and R105Q mutations of PcCHS1 could also change the pH dependence activity as well as the product profile of PcCHS1. Moreover, the Q82P/C198F double mutant could rescue the complete loss of enzyme activity caused by C198F single mutation. Our work also provided an interesting example for the structure‐function relationship studies on CHS.

## Author contributions

HW, YS, XL and TC designed the study. YS and XL carried out experiments or contributed critical reagents and protocols, analyzed the data and performed statistical analyses. HW, YS, XL and TC wrote the manuscript. All the authors read and approved the manuscript.
